# Hand and Forearm Flaps: Maximizing Utility of Cadaveric Specimens

**Published:** 2020-05-27

**Authors:** Mehul Thakkar, James Henderson

**Affiliations:** Department of Plastic Surgery, Southmead Hospital, North Bristol NHS Trust, Westbury-on-Trym, Bristol, United Kingdom

**Keywords:** hand flaps, teaching, cadaveric hand course, local flaps, regional flaps

Dear Sir,

Cadaveric hand surgery courses are increasingly popular amongst plastic surgery and orthopedic trainees in the United Kingdom and beyond. They offer the opportunity to perform complex soft-tissue reconstructions in a pressure-free environment on the closest approximation to living human tissue. Trainees enhance their understanding of surgical approaches and gain confidence in their ability to carry out such procedures in the operating theater.

We must ensure that the maximum possible benefit is obtained from every single specimen while being mindful of the donors’ generosity in leaving themselves to the betterment of medical practice. Cadaveric tissue is too precious a resource to waste.

We identified common and classic flaps in the hand and forearm along with other standard approaches to the hand. We then combined them in to a logical scheme in order to maximize the use of the available tissues.

We present these schemata in Figures 1 and 2. Our plans enable the donor sites of several flaps to be used as defects for subsequent flap reconstructions, enabling the trainee to perform the reconstructive element as well, or the chance to repeat flaps if desired.

We include 22 local/regional flaps and approaches in the scheme for each hand and forearm. The ring and little fingers can also be used for flexor/extensor tendon repairs, nerve repairs, and fracture fixation, further maximizing the training opportunities afforded by each specimen.

## Figures and Tables

**Figure 1:**
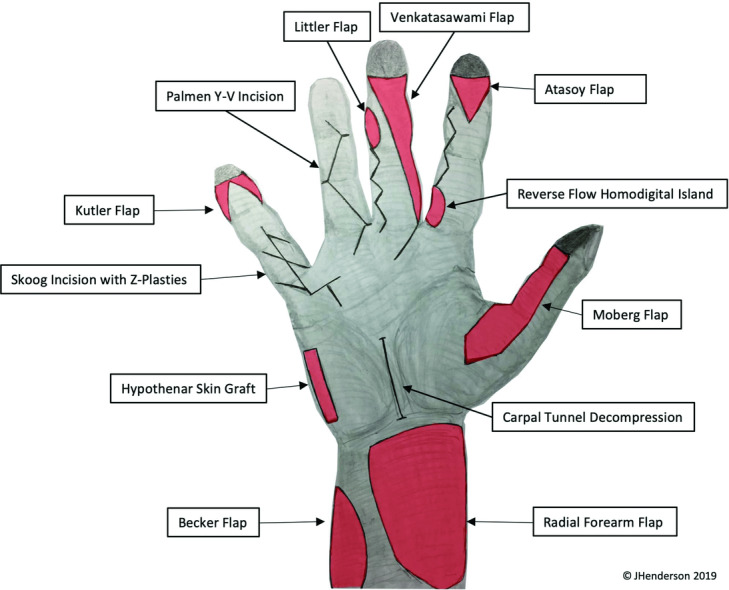
Volar flaps. Used with permission.

**Figure 2:**
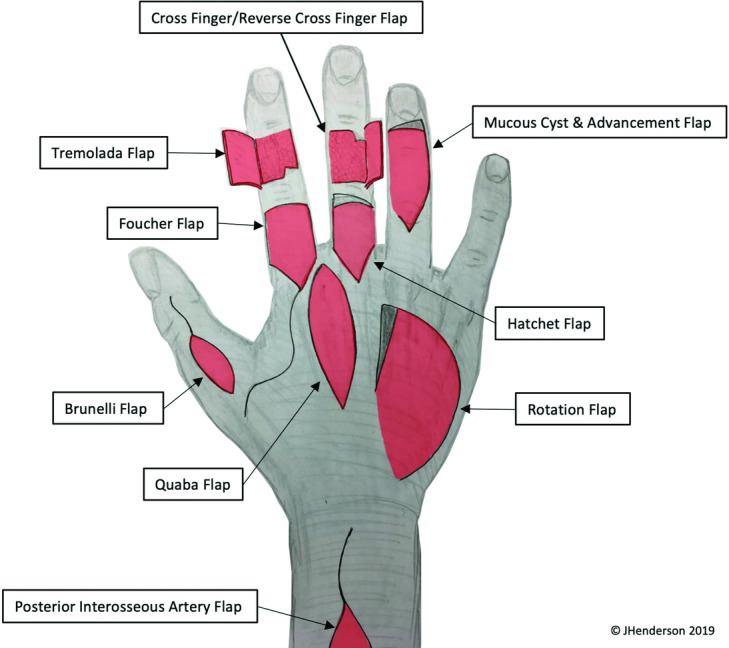
Dorsal flaps. Used with permission.

